# Rupture of ectopic renal arterial pseudoaneurysm after percutaneous nephrolithotomy

**DOI:** 10.1590/S1677-5538.IBJU.2015.0017

**Published:** 2016

**Authors:** Mingshuai Wang, Junhui Zhang, Nianzeng Xing

**Affiliations:** 1 Department of Urology, Beijing Chao - Yang Hospital, Capital Medical University, Beijing, China

## Abstract

A 35-year-old female patient presented with swelling pain at left waist for 1 month. Left renal pelvis stones were found and standard percutaneous nephrolithotomy was successfully performed. Two weeks later, the patient suddenly suffered massive bleeding presented with gross hematuria. Rupture of ectopic renal artery pseudoaneurysm was identified by computed tomography and angiography of the renal artery. Emergency selective angioembolization of one branch of the artery was performed. To our knowledge, this is the first report of ruptured ectopic renal arterial pseudoaneurysm.

## DESCRIPTION OF CASE

**Figure 1:**
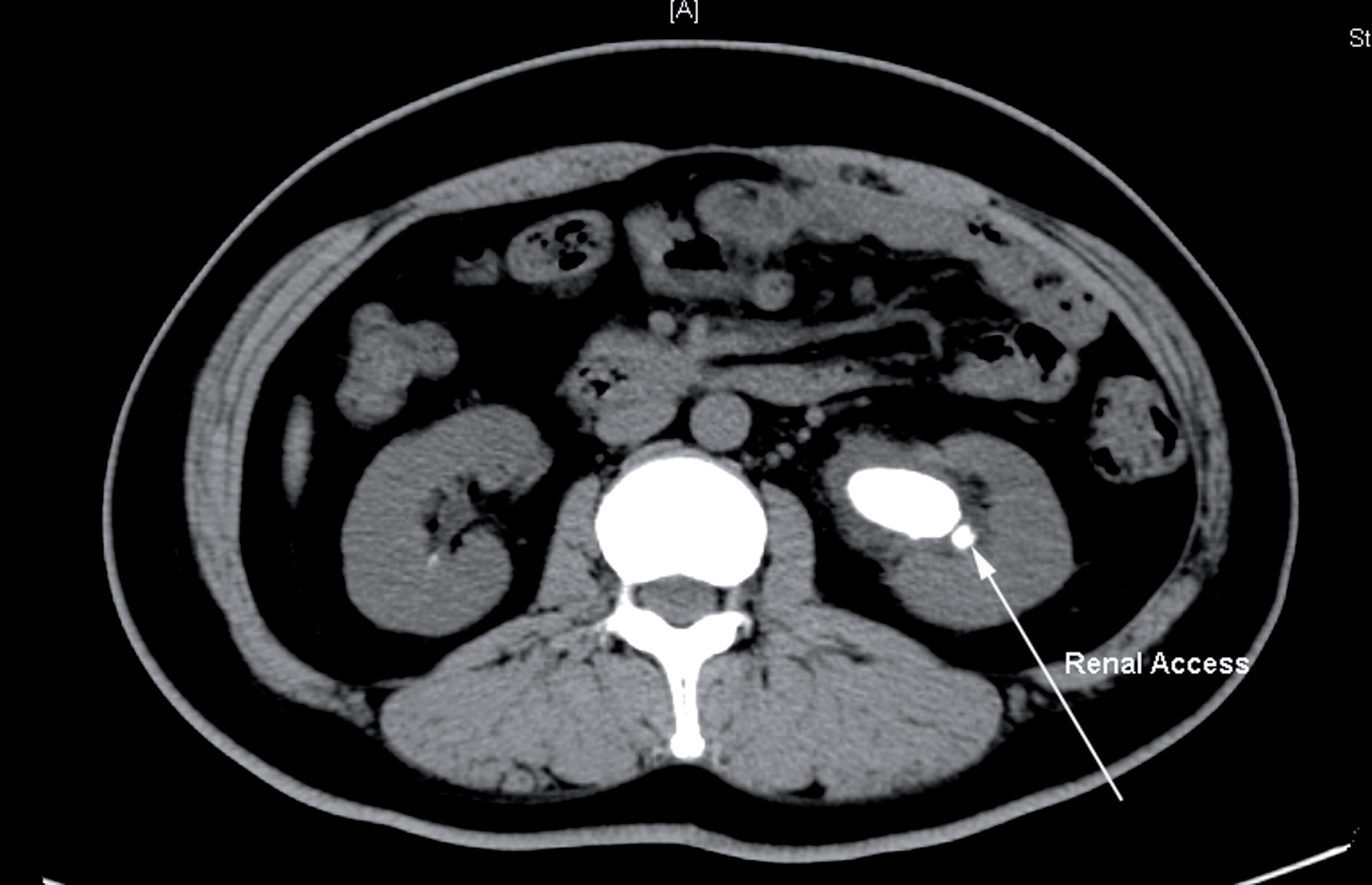
Left renal stone, measuring 3.2*1.6cm.

A 35-year-old female patient presented with back pain for 1 month. Plain computed tomography (CT) scan showed a stone measuring 3.2*1.6cm and a smaller one located in the left renal pelvis (Figure-1).

**Figure 2:**
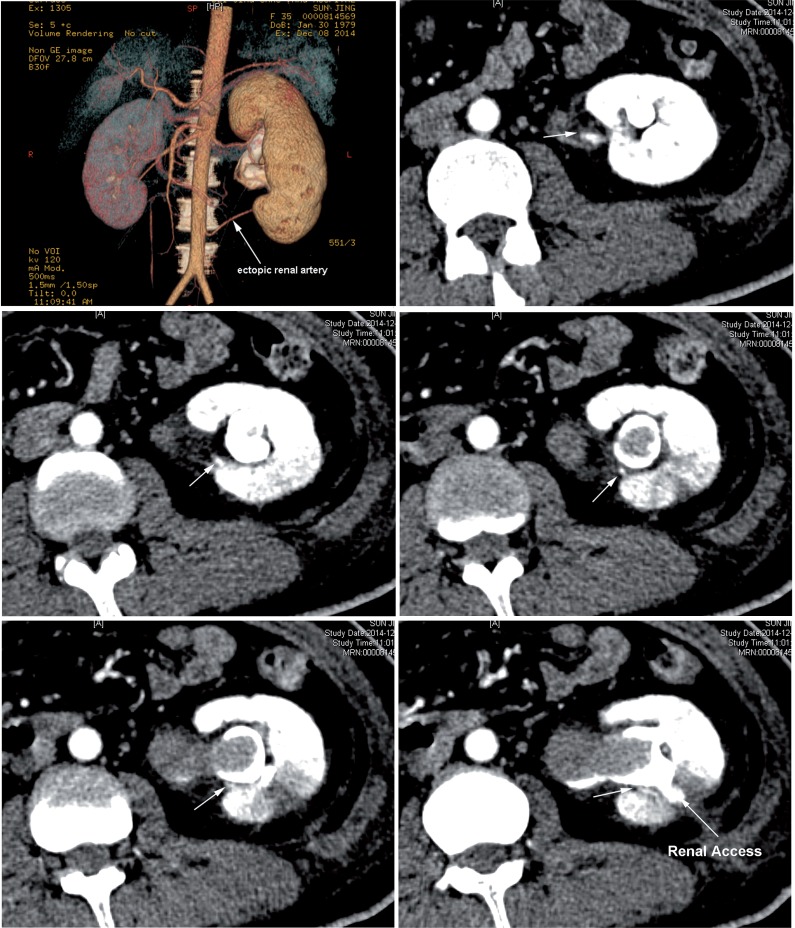
One ectopic renal artery was found in CT angiography of the left renal artery, and a branch of this artery was near the renal access.

**Figure 3:**
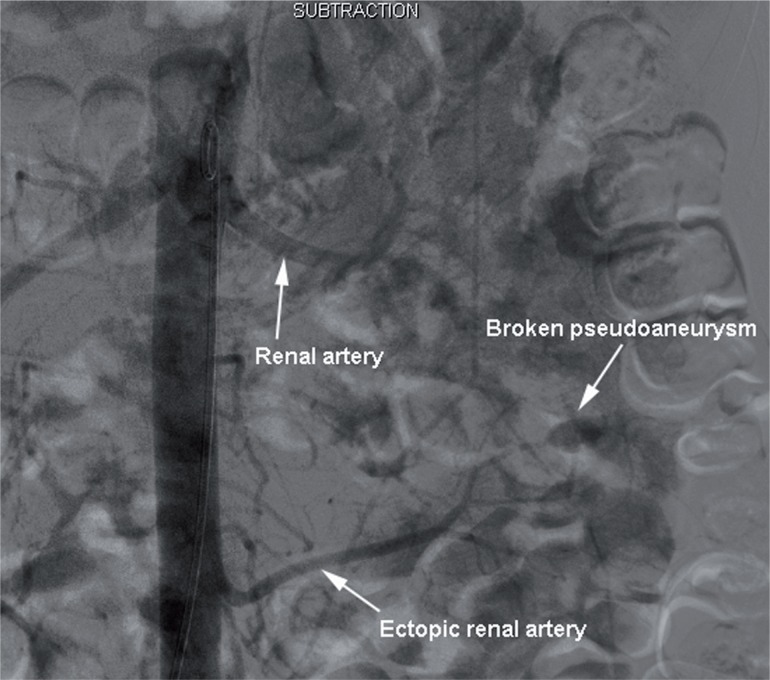
Rupture of one branch of the ectopic renal artery angiography.

One experienced surgeon performed standard percutaneous nephrolithotomy. After general anesthesia, percutaneous renal access was obtained under ultrasound with an 18-gauge needle. Tract dilatation was accomplished using balloon dilator of 24F. The stone was fragmented utilizing an ultrasonic lithotripter through a rigid 24F nephroscope. A 20F nephrostomy tube was inserted after the successful completion of the procedure. The nephrostomy tube and urinary catheter were removed 1 week postoperatively. Unfortunately, 2 weeks after operation, the patient suddenly suffered massive bleeding presented with gross hematuria. Her blood hemoglobin decreased to 7.2g/L. CT angiography identified an ectopic renal artery leading to a pseudoaneurysm which appeared to be in the pathway of the access tract (Figure-2). Emergency selective angioembolization of this branch was performed in conjunction with the angiogram confirming rupture and pseudoaneurysm of the ectopic branch artery (Figure-3).

Arterial pseudoaneurisms have occurred as consequence of extracorporeal shock wave lithotripsy (1). Gavant et al. firstly described rupture of renal pseudoaneurysm as a complication of percutaneous nephrostomy (2). It was reported that pseudoaneurysm after percutaneous renal surgery was the most common angiographic finding (3). The access route to the stone has a major impact on the incidence of the complication, causing pseudo-aneurisms or arterio-venous fistulae. Because of the trajectory of the access-tract between arterial and venous channels in the upper and mid-pole arterial-venous fistula-formation may occur. This trajectory is different for a subcostal and intercostal approach, hence a different rate of attendant complications (4). However, rupture of ectopic renal arterial pseudoaneurysm is very rare, so much care should be taken for this kind of patient.
